# Molecular Characterization of *Clostridium difficile* Isolates from Human Subjects and the Environment

**DOI:** 10.1371/journal.pone.0151964

**Published:** 2016-03-24

**Authors:** Tian-tian Tian, Jian-hong Zhao, Jing Yang, Cui-xin Qiang, Zhi-rong Li, Jing Chen, Kai-yue Xu, Qing-qing Ciu, Ru-xin Li

**Affiliations:** 1 Department of Clinical Microbiology, Second Hospital of Hebei Medical University, Hebei Provincial Center for Clinical Laboratories, Shijiazhuang City, Hebei Province, China; 2 Health Care Department, Maternal and Child Health Care Center of Cangzhou, Cangzhou City, Hebei Province, China; Cleveland Clinic, UNITED STATES

## Abstract

*Clostridium difficile* is a spore-forming, gram-positive, anaerobic bacillus that can cause *C*. *difficile* infection (CDI). However, only a few studies on the prevalence and antibiotic resistance of *C*. *difficile* in healthy individuals in China have been reported. We employed a spore enrichment culture to screen for *C*. *difficile* in the stool samples of 3699 healthy Chinese individuals who were divided into 4 groups: infants younger than 2 years of age and living at home with their parents; children aged 1 to 8 years of age and attending three different kindergarten schools; community-dwelling healthy adult aged 23–60 years old; and healthcare workers aged 28–80 years old. The *C*. *difficile* isolates were analyzed for the presence of toxin genes and typed by PCR ribotyping and multilocus sequence typing (MLST). The minimum inhibitory concentration of 8 antimicrobial agents was determined for all of the isolates using the agar dilution method. The intestinal carriage rate in the healthy children was 13.6% and ranged from 0% to 21% depending on age. The carriage rates in the 1654 community-dwelling healthy adults and 348 healthcare workers were 5.5% and 6.3%, respectively. Among the isolates, 226 were toxigenic (225 *tcdA*+/*tcdB*+ and 1 *tcdA*+/*tcdB*+ *ctdA*+/*ctdB*+). Twenty-four ribotypes were found, with the dominant type accounting for 29.7% of the isolates. The toxigenic isolates were typed into 27 MLST genotypes. All of the strains were susceptible to vancomycin, metronidazole, fidaxomicin, and rifaximin. High resistance to levofloxacin and ciprofloxacin at rates of 39.8% and 98.3%, respectively, were observed. ST37 isolates were more resistant to levofloxacin than the other STs. The PCR ribotypes and sequence types from the healthy populations were similar to those from the adult patients.

## Introduction

*Clostridium difficile* is a spore-forming, gram-positive, anaerobic bacillus that mainly spreads via the fecal-oral route and can cause *C*. *difficile* infection (CDI). The main pathogenic mechanism is the production of enterotoxin A and cytotoxin B [1]. The clinical presentation of infection may range from asymptomatic colonization and self-limiting diarrhea to pseudomembranous colitis and even death [2]. In healthy adults, the rate of *C*. *difficile* colonization is 1–7%, whereas in infants, the rate of asymptomatic colonization can be as high as 2–75% [3–11]. *C*. *difficile* spores can survive for many months in the environment because they are resistant to most disinfectants, heat, and desiccation [12]. It is generally believed that CDI is related to antibiotics use and hospitalization history. However, recent studies have shown that community-acquired CDI is increasing, especially in younger individuals without a history of antibiotic use [13–17]. Studies on the prevalence of *C*. *difficile* in healthy populations have been conducted in a number of countries [3, 10, 18]; however, few reports from China are available. Multiple studies have demonstrated that healthy individuals in a community may constitute a possible reservoir for community-acquired CDI [3, 19]. However, because of differences in geographical location and lifestyle, it remains unclear whether asymptomatic carriers play a role in these infections. This study investigated the prevalence and molecular characterization of *C*. *difficile* among 3699 healthy individuals in China. We also examined the antimicrobial susceptibility patterns of *C*. *difficile* colonization in healthy individuals. This study aimed to perform the first comprehensive epidemiological study of *C*. *difficile* colonization in healthy populations in China.

## Materials and Methods

### Study subjects

In total, 3699 healthy individuals were tested for the intestinal carriage of *C*. *difficile* by stool culture between September 2013 and September 2014. Individuals who reported diarrhea, hospitalization, surgery, *C*. *difficile* infection or exposure to antimicrobial agents in the 8 weeks prior to the study were excluded. Stool specimens were collected from each individual for isolation of *C*. *difficile*.The subjects were divided into groups A, B, and C according to their age and surrounding environment. Group A consisted of 36 infants younger than 2 year of age who live at home with their parents. Group B included 1661 children aged 1–8 years who attend 3 kindergartens located in Xingtai City (kindergarten I) and Cangzhou City (kindergarten II and III) of Hebei Province. These cities are separated by more than 300 kilometers. Group C consisted of 1654 community-dwelling healthy adults (male/female = 0.84) aged 23–60 years old. The healthcare workers were further divided into a separate group because of their occupation. Therefore, Group D included 348 healthcare workers (male/female = 0.61) from the Second Hospital of Hebei Medical University aged 28–80 years old. This study was approved by the Second Hospital of Hebei Medical University Institutional review board [approval no.2013L-18]. Written informed consent was obtained from all of the participants and guardians of the children enrolled in the study.

### Clinical data collection

Clinical data were collected using a questionnaire. For all 36 infants, the sex, age, date of birth, delivery route, and feeding characteristics (breast milk fed or formula milk fed and diversity of specific food types, including eggs, millet gruel and floss) were noted in the questionnaire. For the other subjects, sex and age data were collected. To provide a comparison with the isolates from patients in a hospital environment, *C*. *difficile* isolates from 383 CDI inpatients in the Second Hospital of Hebei Medical University that were evaluated in our previous study were used in this study. Additionally, 33 *C*. *difficile* isolates recovered from adult inpatients with diarrhea in 2011 and analyzed with PCR ribotyping and multilocus sequence typing (MLST) were included in this study[20].

### Isolation and identification of *C*. *difficile*

Stool samples were incubated in *C*. *difficile* moxalactam-norfloxacin (CDMN, Oxoid, Cambridge, UK) broth supplemented with 0.1% sodium taurocholate at 37°C for 5 days. The culture samples were mixed with equal volumes of absolute ethanol and incubated at room temperature for 60 min. The samples were centrifuged at 3000 ×g for 10 min, and 500 μL of the pellet was plated on *C*. *difficile* selective medium (Oxoid). The plates were incubated in anaerobic conditions at 37°C for 48 h. Presumptive *C*. *difficile* colonies were identified based on their morphology and odor using a Vitek 2 ANC card (bioMérieux, France). The isolated strains were stored in 15% (v/v) glycerol at -80°C.

### DNA extraction

The bacteria stored in 15% (v/v) glycerol were transferred to a blood plate, and *C*. *difficile* was grown on blood agar for 48 h. Three colonies were suspended in 1 mL of sterile ultrapure deionized water, and DNA was extracted using a genomic DNA purification kit (TIANamp Bacteria DNA Kit; Tiangen Biotech, Beijing, China) according to the manufacturer’s instructions. DNA samples were stored at -20°C until use.

### Detection of *tcdA*, *tcdB*, and binary toxin genes

The *tcdA*, *tcdB*, *cdtA*, and *cdtB* toxin genes and 16S rDNA were analyzed by 5-plex PCR as described by Persson et al. [21]. The PCR reactions were conducted in a reaction volume of 25 μl that consisted of 12.5 μL of 2× Taq MasterMix, 2.25 μL of double-distilled H_2_O, 2 μL of DNA, and 8.25 μL of primers (Table 1). The thermal cycling conditions were as follows: 94°C for 10 min; 30 cycles of 94°C for 50 s, 52°C for 50 s, and 72°C for 50 s; and a final extension at 72°C for 10 min. The PCR products were analyzed by electrophoresis on a 1.5% agarose gel.

**Table 1:** Primers used in the present study.

Analysis	Gene target	Primer name	Sequence (5'–3')	Amplicon size (bp)
5-plex PCR	tcdA	tcdA-F3345	GCATGATAAGGCAACTTCAGTGGTA	629
		tcdA-R3969	AGTTCCTCCTGCTCCATCAAATG	
	tcdB	tcdB-F5670	CCAAARTGGAGTGTTACAAACAGGTG	410
		tcdB-R6079A	GCATTTCTCCATTCTCAGCAAAGTA	
		tcdB-R6079B	GCATTTCTCCGTTTTCAGCAAAGTA	
	cdtA	cdtA-F739A	GGGAAGCACTATATTAAAGCAGAAGC	221
		cdtA-F739B	GGGAAACATTATATTAAAGCAGAAGC	
		cdtA-R958	CTGGGTTAGGATTATTTACTGGACCA	
	cdtB	cdtB-F617	TTGACCCAAAGTTGATGTCTGATTG	262
		cdtB-R878	CGGATCTCTTGCTTCAGTCTTTATAG	
	16S rDNA	PS13	GGAGGCAGCAGTGGGGAATA	1062
		PS14	TGACGGGCGGTGTGTACAAG	
PCR ribotyping	16–23S rDNA	PRB	GTGCGGCTGGATCACCTCCT	
		PRBas	CCCTGCACCCTTAATAACTTGACC	
MLST	adk	adk1F	TTACTTGGACCTCCAGGTGC	635
		adk1R	TTTCCACTTCCTAAGGCTGC	
	atpA	atpA1F	TGATGATTTAAGTAAACAAGCTG	674
		atpA1R	AATCATGAGTGAAGTCTTCTCC	
	dxr	dxr3F	GCTACTTTCCATTCTATCTG	525
		dxr4R	CCAACTCTTTGTGCTATAAA	
	glyA	glyA1F	ATAGCTGATGAGGTTGGAGC	625
		glyA1R	TTCTAGCCTTAGATTCTTCATC	
	recA	recA2F	CAGTAATGAAATTGGGAGAAGC	705
		recA2R	ATTCAGCTTGCTTAAATGGTG	
	sodA	sodA5F	CCAGTTGTCAATGTATTCATTTC	585
		sodA6R	ATAACTTCATTTGCTTTTACACC	
	tpi	tpi2F	ATGAGAAAACCTATAATTGCAG	640
		tpi2R	TTGAAGGTTTAACACTTCCACC	

### PCR ribotyping

Specific oligonucleotide primers complementary to the 3' end of the 16S rRNA gene and the 5' end of the 23S rRNA gene were used to amplify the variable-length intergenic spacer region (Table 1). Amplification was performed as described by Bidet et al. [22]. The PCR products were separated by electrophoresis on a 3% agarose gel for 4 h at 100 V, and the PCR ribotyping (PR) profiles were analyzed using Quantity One software (Bio-Rad, California, USA). A 100-bp DNA ladder was used to normalize the profiles, and reference strain 027(ATCC BAA-1870) was used in this study. Brazier’s nomenclature was used to classify the PR profiles. In other cases, the HB prefix followed by our laboratory was adopted according to the order in which the profiles appear.

### MLST

MLST was performed as described by Griffiths et al. [23]. The following 7 control genes were targeted: *adk*, *atpA*, *dxr*, *glyA*, *recA*, *sod*, and *tpi* (Table 1). The amplified products were sent to Sangon Biotech (Shanghai, China) and the Beijing Genomics Institute (Beijing, China) for sequencing. The DNA sequences of the 7 genes were submitted to the MLST database (http://pubmlst.org/clostridiumdifficile) to obtain the sequence type (ST).

### Antimicrobial susceptibility testing

The minimum inhibitory concentrations (MICs) of vancomycin, metronidazole, fidaxomicin, rifaximin, levofloxacin, ciprofloxacin, meropenem, and chloramphenicol were determined using the agar dilution method recommended by the Clinical and Laboratory Standards Institute (CLSI) [24]. The MIC is defined as the lowest concentration of an antimicrobial agent that inhibits the growth of a tested isolate. *C*. *difficile* ATCC 700057 was used for quality control in each run of the susceptibility testing. The MIC interpretive breakpoints for metronidazole, levofloxacin, ciprofloxacin, meropenem, and chloramphenicol were chosen according to the CLSI guidelines, and the breakpoints for vancomycin (susceptible, MIC of 2 μL/mL; and resistant, MIC of > 2 μL/mL) were those recommended by The European Committee on Antimicrobial Susceptibility Testing (EUCAST) [25]. Breakpoints were not established for rifaximin and fidaxomicin.

### Statistical analysis

All of the statistical analyses were performed using SPSS version 13.0 for Windows (SPSS, Chicago, IL, USA). The χ^2^ test or Fisher’s exact test was performed to analyze categorical variables. The level of statistical significance was defined as *P* < 0.05.

## Results

### Prevalence of intestinal colonization

Of the 36 infants in Group A, 9 (25%) carried *C*. *difficile*, with 7 (19.4%) carrying toxigenic (*tcdA*+/*tcdB*+) isolates. Factors that may have influenced *C*. *difficile* colonization were analyzed for these 36 infants (Table 2).

**Table 2:** Characteristics of infants aged 0–2 years with and without *C*. *difficile*.

Characteristic	*C*. *difficile* carriers (n = 9)	*C*. *difficile* non-carriers (n = 27)	*P* ^a^
Sex, M/F	6/3	18/9	1
Median age in months (range)	6 (1–18)	5 (1–24)	0.408
Breast milk feeding	8	25	1
Food diversity at time of stool collection	7	9	0.049
Delivery route (vaginal/caesarean)	2/7	15/12	0.128

^a^ Comparisons between carriers and non-carriers of *C*. *difficile* were performed using Fisher’s exact test.

Table 3 shows the number of *C*. *difficile* strains isolated from the 1661 children in the 3 kindergartens (male:female = 1.34). *C*. *difficile* was isolated from the stool samples of 222 children (13.4%), and 146 (65.8%) of the isolates were toxigenic (*tcdA*+/*tcdB*+). The carriage rates of the children in the I, II, and III kindergartens were 13.6% (127/931), 13.1% (79/600), and 12.3% (16/130), respectively. When analyzed according to age group, the carriage rates were 0%, 13.9%, 18.0%, 11.9%, 12.4%, and 6.3% in the infants and children aged 1, 2, 3, 4, 5 and 6 years, respectively.

**Table 3:** Asymptomatic intestinal *C*. *difficile* carriage rate (%) in healthy children in the 3 kindergartens(group B).

Age (years)	1–2	2–3	3–4	4–5	5–6	6–7	7–8	Total
Kindergarten I	0/3 (0)	8/69 (11.6)	51/288 (17.8)	30/279 (10.8)	35/247 (14.2)	3/38 (7.9)	0/7 (0)	127/931 (13.6)
Kindergarten II	NS	NS	29/144 (20.1)	22/184 (12.0)	26/247 (10.5)	2/25 (8.0)	NS	79/600 (13.1)
Kindergarten III	0/5 (0)	3/10 (30)	3/27 (11.1)	5/16 (31.2)	3/24 (12.5)	2/48 (4.2)	NS	16/130 (12.3)
Total	0/8 (0)	11/79 (13.9)	83/459 (18.0)	57/479 (11.9)	64/518 (12.4)	7/111 (6.3)	0/7 (0)	222/1661 (13.4)

**Note:** NS: no subjects

The prevalence of intestinal carriage among the 1654 community-dwelling healthy adults was 5.5% (91/1654), and 59 of the isolates were *tcdA+/tcdB+* and 1 was *tcdA+/tcdB+ cdtA+/cdtB+*. Of the 348 healthcare workers, 22 were positive for *C*. *difficile*, and 13 of the isolates were *tcdA+/tcdB+*.

### PCR ribotyping

**Fig 1:**
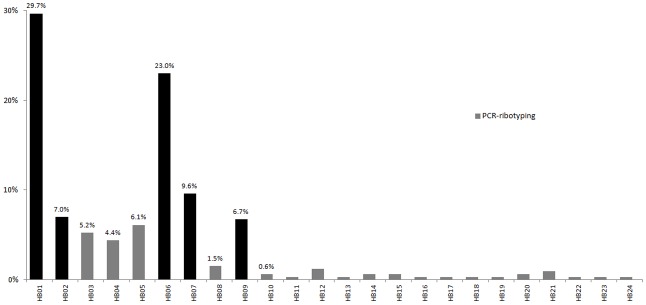
Frequency of PRs among the 344 *C*. *difficile* isolates from healthy Chinese individuals.

In total, 344 *C*. *difficile* strains isolated from healthy individuals were analyzed by PCR ribotyping. Twenty-four PRs were identified, with 5 PRs accounting for 76% (261/344) of the isolates (Fig 1). The most virulent isolate was toxigenic PR027, and it was not identified among the strains from the healthy individuals.

### MLST

**Fig 2:**
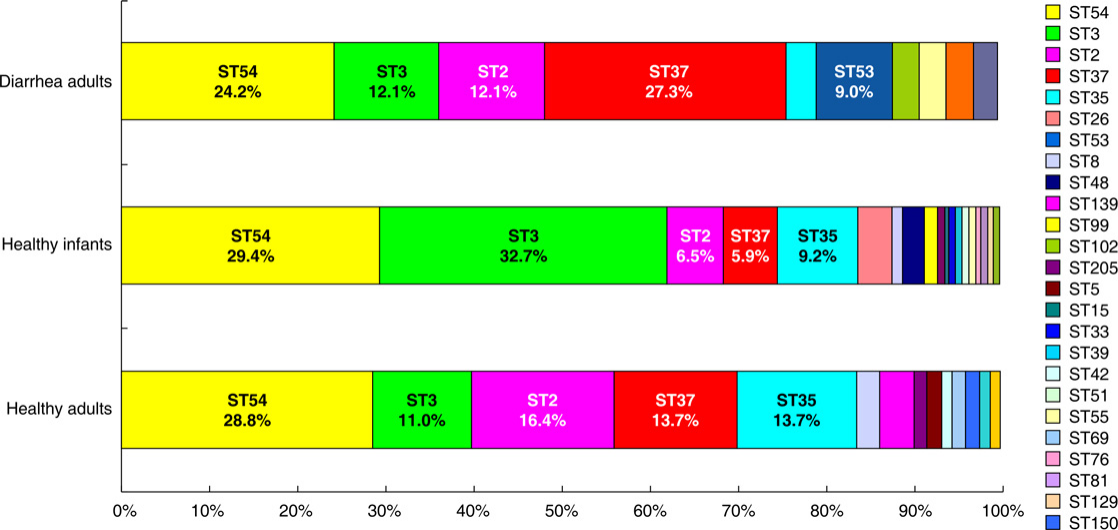
Abundance of STs (%) in the 3 study populations. Data on the STs of *C*. *difficile* isolates from adult inpatients with diarrhea were obtained from a survey conducted in China in 2011.

**Fig 3:**
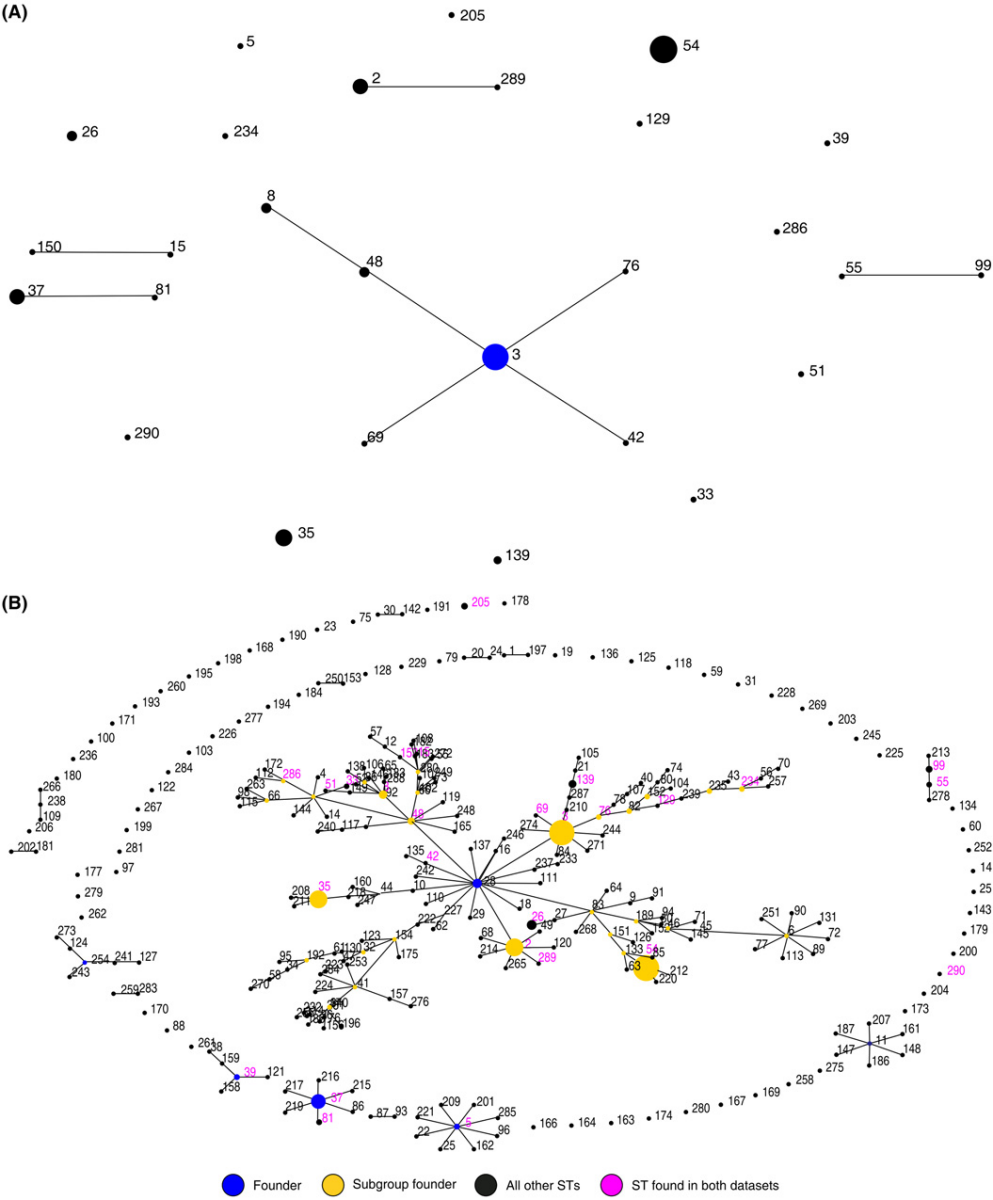
eBURST diagram of the 226 toxigenic *C*. *difficile* isolates from the healthy individuals. (a) Population snapshot showing the clusters of linked sequence types (STs) and unlinked STs in the entire *C*. *difficile* MLST database. (b) Three new STs (286, 289, and 290) were identified in this study.

The 226 toxigenic isolates were typed into 27 MLST genotypes (Fig 2), and ST286, ST289, and ST290 were novel. ST54 (ribotype HB06) was the dominant type and accounted for 29.2% (66/226). The other most frequent types were ST3 (ribotype HB01) (25.7%), ST2 (ribotype HB01) (9.7%), ST35 (ribotype HB03) (10.6%), and ST37 (ribotype HB05) (8.4%). No correlation was found between the host population origin and the genotype. For example, STs 2, 3, 35, 37, 53, 54, and 55 were identified among the isolates from healthy individuals and adult inpatients with diarrhea (Fig 3). The MLST genotypes were analyzed using eBURST version 3 (http://eburst.mlst.net/) and classified into 5 groups. ST3 was identified as the group founder. ST55 and ST99 were in the second group, ST150 and ST15 were in the third group, ST37 and ST81 were in the fourth group, and ST2 and ST289 were in the last group. The other STs (5, 26, 33, 35, 39, 51, 54, 129, 139, 205, 234, 286, and 290) were singletons (Fig 3A). A comparison of our 226 isolates with the isolates in the MLST database showed that the Chinese isolates from healthy individuals and patients were widely scattered over 7 groups (Fig 3B).

### Comparison of *C*. *difficile* strains between healthy individuals and adult patients with CDI

The PRs of the isolates from healthy individuals were compared with those from adult patients with diarrhea. The PRs of the isolates from the patients overlapped well with those from the healthy individuals except for 1 isolate of the non-toxigenic strain HB28, which was found in 1 patient. In addition, the MLST genotypes of the isolates from healthy individuals were compared with those of 33 *C*. *difficile* strains from adult patients with diarrhea caused by CDI. The STs 2, 3, 35, 37, 53, 54, and 55 were identified in the isolates from both the healthy population and the patients, suggesting that the same strains circulated in patients and healthy individuals.

### Antimicrobial susceptibility

All of the strains were susceptible to vancomycin, metronidazole, fidaxomicin, and rifaximin. However, high resistance to levofloxacin and ciprofloxacin was observed, with resistance rates of 39.8% and 98.3%, respectively (Table 4). Additionally, we found that 1.45% and 2% of the *C*. *difficile* isolates were resistant to meropenem and chloramphenicol, respectively, and multiple *tcdA*+/*tcdB*+ isolates were resistant to ciprofloxacin (*P*<0.05, Table 5). The strains isolated from hospitalized patients exhibited higher resistance to levofloxacin and rifaximin compared with those from the healthy population (*P*<0.05). All of the isolates were susceptible to chloramphenicol except for 17% of the ST35 strains (Table 6). Isolates with levofloxacin resistance were identified in ST37 (84%), ST35 (50%), ST54 (39%), and ST2 (32%). Furthermore, ST37 was the most dominant type in the patients with CDI but was limited in healthy individuals.

**Table 4:** MICs of 8 antimicrobial agents for 344 *C*. *difficile* isolates determined by the agar dilution method.

Antimicrobial agent	MIC breakpoint (mg/L)	MIC_50_ (mg/L)	MIC_90_ (mg/L)	Range (mg/L)	Resistance (%)
Vancomycin	> 2	0.25	0.5	0.03–1	0
Metronidazole	≥ 32	0.25	0.5	0.03–1	0
Fidaxomicin^a^		0.06	0.125	0.0075–0.5	
Rifaximin	≥ 4	0.015	0.015	0.0009–0.03	0
Levofloxacin	≥ 8	4	8	2–128	39.8
Ciprofloxacin	≥ 8	8	16	4–128	98.3
Meropenem	≥ 16	2	4	1–16	1.45
Chloramphenicol	≥ 32	8	16	2–64	2.0

^a^ Breakpoint was not established for fidaxomicin.

**Table 5:** MICs of 8 antimicrobial agents for toxigenic and non-toxigenic *C*. *difficile* isolates as determined by using agar dilution.

Antimicrobial agent	*tcdA*+/*tcdB*+ (n = 226 isolates)				*tcdA*-/*tcdB*- (n = 118 isolates)			
	Range (mg/L)	MIC_50_ (mg/L)	MIC_90_ (mg/L)	Resistance (%)	Range (mg/L)	MIC_50_ (mg/L)	MIC_90_ (mg/L)	Resistance (%)
Vancomycin	0.03–1	0.25	0.5	0	0.03–1	0.25	0.5	0
Metronidazole	0.06–0.5	0.25	0.5	0	0.03–1	0.25	0.5	0
Fidaxomicin	0.0075–0.5	0.06	0.125	0	0.0075–0.5	0.06	0.125	0
Rifaximin	0.0009–0.25	0.015	0.015	0	0.0009–0.25	0.015	0.03	0
Levofloxacin	2–128	4	8	40.3	2–128	4	8	39.8
Ciprofloxacin^a^	4–128	16	16	99.6	4–128	8	16	95.8
Meropenem	1–16	2	4	1.8	1–16	2	4	0.8
Chloramphenicol	2–64	8	16	3.5	4–64	8	16	0.8

^a^ MICs between toxigenic and non-toxigenic *C*. *difficile* isolates were compared using Fisher’s exact test (*P* < 0.05).

**Table 6:** Characteristics of the 226 toxigenic *C*. *difficile* strains: genotype and antimicrobial susceptibility.

No.of isolates (%)(total n = 226)	ST	Vancomycin (% R)	Metronidazole (% R)	Fidaxomicin (%R)	Rifaximin (% R)	Levofloxacin (% R)	Ciprofloxacin (% R)	Meropenem (%R)	Chloramphenicol (% R)
66 (29.2)	54^a^	0	0	0	0	39	100	0	3
58 (25.6)	3	0	0	0	0	24	100	3.4	2
24 (10.6)	35	0	0	0	0	50	100	0	17
22 (9.7)	2	0	0	0	0	32	100	4.5	5
19 (8.4)	37	0	0	0	0	84^b^	100	0	5
6 (2.7)	26	0	0	0	0	1/6	0	0	0
4 (1.8)	8	0	0	0	0	0	4/4	0	0
4 (1.8)	48	0	0	0	0	2/4	4/4	0	0
3 (1.3)	139	0	0	0	0	3/3	3/3	0	0
2 (0.9)	205	0	0	0	0	2/2	2/2	0	0
2 (0.9)	99	0	0	0	0	0	2/2	0	0
1 (0.4)	5	0	0	0	0	0	1/1	0	0
1 (0.4)	15	0	0	0	0	1/1	1/1	0	0
1 (0.4)	33	0	0	0	0	0	1/1	0	0
1 (0.4)	39	0	0	0	0	1/1	1/1	0	0
1 (0.4)	42	0	0	0	0	1/1	1/1	0	0
1 (0.4)	51	0	0	0	0	1/1	1/1	0	0
1 (0.4)	55	0	0	0	0	0	1/1	0	0
1 (0.4)	69	0	0	0	0	1/1	1/1	0	0
1 (0.4)	76	0	0	0	0	0	1/1	1/1	0
1 (0.4)	81	0	0	0	0	1/1	1/1	0	0
1 (0.4)	129	0	0	0	0	0	1/1	0	0
1 (0.4)	150	0	0	0	0	1/1	1/1	0	0
1 (0.4)	234	0	0	0	0	1/1	1/1	0	0
1 (0.4)	286	0	0	0	0	0	1/1	0	0
1 (0.4)	289	0	0	0	0	0	1/1	0	0
1 (0.4)	290	0	0	0	0	0	0	0	0

^a^ For ST54, ST3, ST35, ST2, and ST37, resistance is expressed as a %; for the remaining STs, the number of resistant isolates is shown.

^b^ Resistance among the different STs was compared using Fisher’s exact test (vs. levofloxacin, *P* < 0.05)

## Discussion

The stools of healthy individuals were cultured in this study.We look for the *C*. *difficile* carriage rates in healthy children, adults and healthcare workers in China, which were 13.6%, 5.5% and 6.3%, respectively. In addition, a molecular characterization of the *C*. *difficile* isolates identified 24 ribotypes and typed toxigenic isolates into 27 MLST genotypes. All of the isolates were susceptible to vancomycin, metronidazole, fidaxomycin and rifaximin. To our knowledge, this is the first comprehensive epidemiological study of colonized *C*. *difficile* from healthy Chinese populations.

In the present study, the average carriage rate of *C*. *difficile* in healthy Chinese children was 13.6%, and the rate ranged from 6.3% to 21.2% depending on age. The observed carriage rate for infants under 1 year of age was 21%, which was lower than that in other reports. Yamamoto-Osaki et al. [11] conducted a survey at a day nursery and found that in infants younger than 1 year, the carriage rate was 65%. Rousseau et al. [18] found that the carriage rate of *C*. *difficile* was 35% for infants under 1 year and 72% for infants aged 7–9 months. Matsuki et al. [10] reported that the carriage rates for Japanese children aged <1, 1, 2, 3, 4, and 5 years were 100%, 75.0%, 45.5%, 24.0%, 38.5%, and 23.5%, respectively. There may be multiple reasons why the colonization rates in our study were lower. First, it has been suggested that other intestinal microorganisms can inhibit the colonization or growth of *C*. *difficile* [11, 26]. Fallani et al. [27] reported on the fecal microbiota of 605 infants from 5 European countries and found that the fecal microbiota composition varied depending on the geographical origin and feeding method. These findings suggest that the fecal microbiota composition of the Chinese infants studied here may be different from those reported in other countries because of the different diets and geographical origins. The mechanism by which intestinal microorganisms influence *C*. *difficile* colonization must be explored. Second, the *C*. *difficile* carriage status may be transient in infants [28], and because we only collected a single stool sample from each infant, the overall carriage rates may have been underestimated in this study. Third, host and environmental factors may affect intestinal *C*. *difficile* carriage rates. In addition, it has been noted that infants with food diversification have higher rates of *C*. *difficile* colonization compared with infants without food diversification [29]. Furthermore, it has been reported that certain foods, especially meat, contain *C*. *difficile* [30].

A *C*. *difficile* carriage rate of 5.5% was found in the community-dwelling healthy adults, and such data have not been previously reported in China. These results are important for improving our understanding of the prevalence of *C*. *difficile* infection in China and consistent with the results reported by Kato et al. [3]. The PRs from the healthy populations were similar to those from the adult patients, although the HB28 genotype was only identified from adult inpatients with CDI. In addition, Kato et al. [3] identified genotype clusters in community-dwelling healthy adults based on PCR ribotyping and pulsed-field gel electrophoresis genotyping, which suggested that cross-infection can occur among community-dwelling healthy individuals. *C*. *difficile* in community-dwelling healthy adults may play a role in community-acquired CDI; however, the transmission and pathogenic mechanisms are unknown.

Since the outbreak of a hypervirulent strain (ribotype 027, toxinotype III, North American pulsed-field 1 [NAP1]) in North America and several European countries, the incidence and severity of hospital-acquired *C*. *difficile* infections derived from this strain have increased in recent years. In hospital settings, patients are a major source of nosocomial *C*. *difficile* contamination and infection. Healthcare workers are susceptible to colonization and could act as a reservoir for transmission in hospitals. The carriage rates of healthcare workers in previous studies varied, with one study showing no *C*. *difficile* colonization in 55 healthcare workers [31]. In addition, Hell et al. reported the absence of *C*. *difficile* stool carriage in 112 asymptomatic hospital staff in Austria by direct plating onto *C*. *difficile* selective agar (cycloserine/cefoxitin agar) [32]; Kato et al. [3] reported that 4.3% of the hospital staff in Japan carried *C*. *difficile*; and van Nood et al. reported a carriage rate of 13% in healthcare workers in the Netherlands [33]. The hospital staff intestinal carriage rate (6.3%) found in this study was similar to the carriage rate noted for community-dwelling healthy adults. In the present study, the mean age of the participants was 39 years, and they may be representative of a healthy adult population. In addition, several studies have reported that the use of personal protective equipment can reduce the carriage rates of *C*. *difficile*. Johnson and McFarland et al. showed that hand washing and glove wearing reduced the carriage rates to zero [34,35]. Whether carriage rates observed in this study is related to the infection prevention strategies in our hospital is not known, and further research is required. Although hypervirulent strains were not identified in this study, the NAP1 strain has emerged in Asia, and cases were reported in Hong Kong in 2009 and Mainland China in 2014 [36, 37]. Because healthcare workers have frequent and close contact with patients, the use of personal protective equipment and infection prevention strategies for health workers is of paramount importance.

The strains isolated from the healthy population were analyzed by PCR ribotyping and MLST. Three novel STs (ST286, ST289, and ST290) were found in this study. Among the 226 isolates, 22 STs contained a single PR, whereas the remaining 5 STs consisted of 2 or more ribotypes. Certain PRs were associated with more than 1 ST; for example, the HB01 isolates were found in STs 2, 3, and 139. Therefore, the PR of an isolate was not always predictive of the ST for the given isolate, and this result was consistent with the report of Griffiths et al. [23].

The PRs from the healthy populations were similar to those from the adult patients, although the HB28 genotype was only identified from adult inpatients with diarrhea. Furthermore, 50 isolates from healthy children were identified as ST3, which accounted for nearly one third of the 153 healthy children strains but only a small percentage of isolates from the adult patients or healthy individuals. This finding may indicate that the characteristics of the ST3 strain or the children's intestinal environment may facilitate colonization; however, this hypothesis must be further elaborated. ST54 is responsible for many *C*. *difficile* diarrheal infections in Europe, and it was the most frequent type in our study and accounted for 29.2% of isolates from healthy individuals and 24.2% of isolates from CDI patients. These data might provide fundamental insights into the epidemiology of *C*. *difficile* colonization and infection.

All of the tested isolates were sensitive to vancomycin, metronidazole, fidaxomicin, and rifaximin. However, O’Connor et al. [38] reported that rifaximin resistance was common in the BI isolates from a *C*. *difficile* epidemic as determined by restriction endonuclease analysis. In addition, O’Connor et al. [38] suggested that rifaximin resistance is most commonly encountered in *C*. *difficile* isolates after exposure to rifaximin. Our data also demonstrate that strains from patients who had increased exposure to these antimicrobials during hospitalization showed higher resistance to rifaximin compared with isolates from the healthy population. Furthermore, our data showed that the ST37 isolates exhibited a higher rate of resistance to levofloxacin relative to the isolates within the other STs. Additionally, ST37 was the most dominant type in the adult patients with CDI-associated diarrhea, whereas it only had limited prevalence in healthy individuals. This result indicates a relationship between high resistance and pathogenicity in this strain. Further studies are needed to confirm this hypothesis and to investigate the underlying mechanism. Finally, we observed that toxigenic strains are more likely to acquire resistance to ciprofloxacin compared with non-toxigenic strains.

We note certain limitations in this study. First, the principal limitation of this investigation is the small sample size in the group with participants 0–2 years of age, which might have made the colonization rate appear lower. Second, the information was obtained using a questionnaire, which may have produced biased results because of the inclusion of retrospective information.

In summary, to our knowledge, this is the first molecular epidemiological study evaluating the prevalence of *C*. *difficile* in healthy asymptomatic individuals in China. We showed that the carriage rates of *C*. *difficile* in healthy children, community-dwelling healthy adults, and healthcare workers were 13.6%, 5.5%, and 6.3%, respectively. The typing data suggested that certain strains circulate in both healthy individuals and CDI patients, indicating that asymptomatic, colonized individuals may serve as reservoirs for CDI. These results may provide fundamental insights into the prevalence of *C*. *difficile* infections in China.

## References

[1] Geric B, Rupnik M, Gerding DN, Grabnar M, Johnson S. Distribution of *Clostridium difficile* variant toxinotypes and strains with binary toxin genes among clinical isolates in an American hospital. J Med Microbiol, 2004;53: 887–894. 15314196 10.1099/jmm.0.45610-0

[2] Rupnik M, Wilcox MH, Gerding DN. *Clostridium difficile* infection: new developments in epidemiology and pathogenesis. Nat Rev Microbiol, 2009;7: 526–536. 10.1038/nrmicro2164 19528959

[3] Kato H, Kita H, Karasawa T, Maegawa T, Koino Y, Takakuwa H, et al Colonisation and transmission of *Clostridium difficile* in healthy individuals examined by PCR ribotyping and pulsed-field gel electrophoresis. J Med Microbiol. 2001;50: 720–727. 11478676 10.1099/0022-1317-50-8-720

[4] Rexach CE, Tang-Feldman YJ, Cantrell MC, Cohen SH. Epidemiologic surveillance of *Clostridium difficile* diarrhea in a freestanding pediatric hospital and a pediatric hospital at a university medical center. Diagn Microbiol Infect Dis. 2006;56: 109–114. 16678379 10.1016/j.diagmicrobio.2006.03.002

[5] Larson HE, Barclay FE, Honour P, Hill ID. Epidemiology of *Clostridium difficile* in infants. J Infect Dis. 1982;146: 727–733. 7142747 10.1093/infdis/146.6.727

[6] Sandora TJ, Fung M, Flaherty K, Helsing L, Scanlon P, Potter-Bynoe B, et al Epidemiology and risk factors for *Clostridium difficile* infection in children. Pediatr Infect Dis J. 2011;30: 580–584. 10.1097/INF.0b013e31820bfb29 21233782

[7] Collignon A, Ticchi L, Depitre C, Gaudelus J, Delmée M, Corthier G. Heterogeneity of *Clostridium difficile* isolates from infants. Eur J Pediatr. 1993;152: 319–322. 8482281 10.1007/BF01956743

[8] Holst E, Helin I, Mårdh PA. Recovery of *Clostridium difficile* from children. Scand J Infect Dis. 1981;13: 41–45. 7244558 10.1080/00365548.1981.11690365

[9] Penders J, Stobberingh EE, van den Brandt PA, van Ree R, Thijs C. Toxigenic and non-toxigenic *Clostridium difficile*: determinants of intestinal colonisation and role in childhood atopic manifestations. Gut 2008;57: 1025–1026. 10.1136/gut.2007.143214 18559395

[10] Matsuki S, Ozaki E, Shozu M, Inoue M, Shimizu S, Yamaguchi N, et al Colonization by *Clostridium difficile* of neonates in a hospital and infants and children in three daycare facilities of Kanazawa, Japan. Int Microbiol. 2005;8: 43–48. 15906260

[11] Yamamoto-Osaki T, Kamiya S, Sawamura S, Kai M, Ozawa A. Growth inhibition of *Clostridium difficile* by intestinal flora of infant faeces in continuous flow culture. J Med Microbiol. 1994;40: 179–187. 8114067 10.1099/00222615-40-3-179

[12] Weaver L, Michels HT, Keevil CW. Survival of *Clostridium difficile* on copper and steel: futuristic options for hospital hygiene. J Hosp Infect. 2008;68:145–151. 10.1016/j.jhin.2007.11.011 18207284

[13] Centers for Disease Control and Prevention. Severe *Clostridium difficile*-associated disease in populations previously at low risk—four states, 2005. MMWR Morb Mortal Wkly Rep. 2005;54: 1201–1205. 16319813

[14] Khanna S, Pardi DS. The growing incidence and severity of *Clostridium difficile* infection in inpatient and outpatient settings. Expert Rev Gastroenterol Hepatol. 2010;4: 409–416. 10.1586/egh.10.48 20678014

[15] Khanna S, Pardi DS, Aronson SL, Kammer PP, Orenstein R, St Sauver JL, et al The epidemiology of community-acquired *Clostridium difficile* infection: a population-based study. Am J Gastroenterol. 2012;107: 89–95. 10.1038/ajg.2011.398 22108454 PMC3273904

[16] Wilcox MH, Mooney L, Bendall R, Settle CD, Fawley WN. A case-control study of community-associated *Clostridium difficile* infection. J Antimicrob Chemother. 2008;62: 388–396. 10.1093/jac/dkn163 18434341

[17] Bauer MP, Goorhuis A, Koster T, Numan-Ruberg SC, Hagen EC, Debast SB, et al Community-onset *Clostridium difficile*-associated diarrhoea not associated with antibiotic usage—two case reports with review of the changing epidemiology of *Clostridium difficile*-associated diarrhoea. Neth J Med. 2008;66: 207–211. 18490799

[18] Rousseau C, Lemée L, Le Monnier A, Poilane I, Pons JL, Collignon A. Prevalence and diversity of *Clostridium difficile* strains in infants. J Med Microbiol. 2011;60: 1112–1118. 10.1099/jmm.0.029736-0 21393454

[19] Galdys AL, Nelson JS, Shutt KA, et al Prevalence and duration of asymptomatic *Clostridium difficile* carriage among healthy subjects in Pittsburgh, Pennsylvania. J Clin Microbiol. 2014;52(7): 2406–2409. 10.1128/JCM.00222-14 24759727 PMC4097745

[20] Jing Yang, Zhirong Li, Jianhong Zhao, Cuixin Qiang, Hong Li, Hainan Wen, et al Multilocus sequence typing of 33 clinical *Clostridium difficile* isolates. Clin J Lab Med. 2013;36: 920–925

[21] Persson S, Torpdahl M, Olsen KE. New multiplex PCR method for the detection of *Clostridium difficile* toxin A (tcdA) and toxin B (tcdB) and the binary toxin (cdtA/cdtB) genes applied to a Danish strain collection. Clin Microbiol Infect. 2008;14: 1057–1064. 10.1111/j.1469-0691.2008.02092.x 19040478

[22] Bidet P, Barbut F, Lalande V, Burghoffer B, Petit JC. Development of a new PCR-ribotyping method for *Clostridium difficile* based on ribosomal RNA gene sequencing. FEMS Microbiol Lett. 1999;175: 261–266. 10386377 10.1111/j.1574-6968.1999.tb13629.x

[23] Griffiths D, Fawley W, Kachrimanidou M, Bowden R, Crook DW, Fung R, et al Multilocus sequence typing of *Clostridium difficile*. J Clin Microbiol. 2010;48: 770–778. 10.1128/JCM.01796-09 20042623 PMC2832416

[24] Clinical and Laboratory Standards Institute. Methods for antimicrobial susceptibility testing of anaerobic bacteria—seventh edition. Approved standard M11-A7. CLSI, 2010

[25] European Committee on Antimicrobial Susceptibility Testing. 2011. Clinical breakpoint tables, version 1.3. European Committee on Antimicrobial Susceptibility Testing, London, United Kingdom. Available: http://www.eucast.org/eucast_susceptibility_testing/breakpoints/.

[26] McFarland LV, Brandmarker SA, Guandalini S. Pediatric *Clostridium difficile*: a phantom menace or clinical reality? J Pediatr Gastroenterol Nutr. 2000;31: 220–231. 10997362 10.1097/00005176-200009000-00004

[27] Fallani M, Amarri S, Uusijarvi A, Adam R, Khanna S, Aguilera M, et a1. Determinants of the human infant intestinal microbiota after the introduction of first complementary food in infant samples from five European centres. Microbiology. 2011;157: 1385–1392. 10.1099/mic.0.042143-0 21330436

[28] Jangi S, Lamont J. Asymptomatic by *Clostridium difficile* in infants: implications for disease in later life. J Pediatr Gastroenterol Nutr. 2010;51: 2–7. 10.1097/MPG.0b013e3181d29767 20512057

[29] Rousseau C, Poilane I, De Pontual L, Maherault AC, Le Monnier A, Collignon A. *Clostridium difficile* carriage in healthy infants in the community: a potential reservoir for pathogenic strains. Clin Infect Dis. 2012;55: 1209–1215. 10.1093/cid/cis637 22843784

[30] Bouttier S, Barc MC, Felix B, Lambert S, Collignon A, Barbut F. *Clostridium difficile* in ground meat, France. Emerg Infect Dis. 2010;16: 733–735. 10.3201/eid1604.091138 20350408 PMC3321948

[31] Carmeli Y, Venkataraman L, DeGirolami PC, Lichtenberg DA, Karchmer AW, Samore MH. Stool colonization of healthcare workers with selected resistant bacteria. Infect Control Hosp Epidemiol. 1998;19: 38–40. 9475348 10.1086/647705

[32] Hell M, Sickau K, Chmelizek G, Kern JM, Maass M, Huhulescu S, et al Absence of *Clostridium difficile* in asymptomatic hospital staff. Am J Infect Control 2012;40: 1023–1024. 10.1016/j.ajic.2012.01.018 22572458

[33] Van Nood E, van Dijk K, Hegeman Z, Speelman P, Visser CE. Asymptomatic carriage of *Clostridium difficile* among HCWs: do we disregard the doctor? Infect Control Hosp Epidemiol. 2009;30: 924–925. 10.1086/605642 19653823

[34] Johnson S, Gerding DN, Olson MM, et al Prospective, controlled study of vinyl glove use to interrupt Clostridium difficile nosocomial transmission. Am J Med. 1990;88:137–140. 2301439 10.1016/0002-9343(90)90462-m

[35] McFarland LV, Mulligan ME, Kwok RY, Stamm WE. Nosocomial acquisition of *Clostridium difficile* infection. N Engl J Med. 1989;320:204–210. 2911306 10.1056/NEJM198901263200402

[36] Wang P, Zhou Y, Wang Z, Xie S, Zhang T, Lin M, et al Identification of *Clostridium difficile* ribotype 027 for the first time in Mainland China. Infect Control Hosp Epidemiol. 2014;35: 95–98. 10.1086/674405 24334809

[37] Cheng VC, Yam WC, Chan JF, To KK, Ho PL, Yuen KY. *Clostridium difficile* ribotype 027 arrives in Hong Kong. Int J Antimicrob Agents 2009;34: 492–493. 10.1016/j.ijantimicag.2009.04.004 19464857

[38] O’Conor JR, Galang MA, Samblo SP, Hecht DW, Vedantam G, Gerding DN, et al Rifampin and rifaximin resistance in clinical isolates of *Clostridium difficile*. Antimicrob Agents Chemother. 2008;52: 2813–2817. 10.1128/AAC.00342-08 18559647 PMC2493101

